# Geriatric nutritional risk index as readmission predictor in older adults with heart failure irrespective of ejection fraction

**DOI:** 10.20407/fmj.2022-028

**Published:** 2022-12-27

**Authors:** Yoshihiro Sato, Masahiro Kumada, Hideki Kawai, Sadako Motoyama, Masayoshi Sarai, Tsutomu Nakagawa, Hideo Izawa

**Affiliations:** 1 Department of Cardiology, Fujita Health University, School of Medicine, Toyoake, Aichi, Japan; 2 Division of Cardiology, Toyonaka Municipal Hospital, Toyonaka, Osaka, Japan

**Keywords:** Malnutrition, Acute decompensated heart failure, Objective nutritional index, Readmission

## Abstract

**Objectives:**

Malnutrition is associated with an increased risk of hospital readmission for heart failure in patients with acute decompensated heart failure (ADHF). Therefore, evaluation of the nutritional status in patients with ADHF may be important. The geriatric nutritional risk index (GNRI), the controlling nutritional status (CONUT) score, and the prognostic nutritional index (PNI) are widely used objective indexes for evaluation of the nutritional status. The present study was performed to determine the best nutritional index for predicting the prognosis in older adults with ADHF.

**Methods:**

We retrospectively studied 167 older adults (>65 years of age) who were admitted with ADHF from January 2012 to December 2015 and discharged alive. The objective nutritional status was evaluated using the GNRI, CONUT score, and PNI at admission. The endpoint of this study was unplanned hospitalization for worsening heart failure (WHF) within 1 year after discharge.

**Results:**

During the follow-up period, 58 patients were readmitted for WHF. In the multivariate Cox analysis, only the GNRI (*p*<0.0001) was independently associated with readmission for WHF among the three nutritional indexes. Kaplan–Meier analysis revealed that patients in the low-GNRI group (<90 as determined by receiver operating characteristic curve analysis) had a significantly greater risk of 1-year hospital readmission for WHF (*p*<0.0001; hazard ratio, 6.1; 95% confidence interval, 3.5–10.5).

**Conclusion:**

Among the objective nutritional indexes, the GNRI is the best predictor of readmission for WHF within 1 year after discharge in older adults with ADHF.

## Introduction

Despite the widespread use of standard medical treatment and device therapy for heart failure based on clinical evidence, the prognosis of patients with heart failure requires improvement. The number of patients with heart failure is steadily increasing worldwide because of the increase in the aging population, which has led to major increases in the medical and economic burdens on society. Although several reports have revealed that the rate of heart failure with a preserved ejection fraction (HFpEF) among older adults is especially high, there is a distinct lack of evidence for prediction of the clinical course and prognosis.^1^ Approximately 27% to 47% of older adults who present with acute decompensated heart failure (ADHF) are readmitted to the hospital within 3 to 6 months after discharge,^2^ resulting in major increases in medical costs and poor outcomes, especially a decreased quality of life. Therefore, early risk stratification and treatment of patients with heart failure are important to prevent readmission.^3^

Malnutrition in patients with heart failure is one of the signs of cardiac cachexia and is closely associated with poor outcomes.^4–6^ A method with which to appropriately and easily evaluate patients’ nutritional status in the daily clinical setting is important.^7^ At present, the geriatric nutritional risk index (GNRI), the controlling nutritional status (CONUT) score, and the prognostic nutritional index (PNI) are widely used as clinical indicators of the nutritional status. These indexes are calculated on the basis of body weight (BW), serum albumin level, serum total cholesterol level, and total lymphocyte count. Recent studies have shown that all three indexes are useful to predict the prognosis of patients with heart failure.^8–10^ However, there are no data regarding which index is the most predictive of rehospitalization for heart failure in patients who were admitted with ADHF, especially patients of advanced age (>65 years).

The purpose of our study was to determine which nutritional index is most closely associated with the risk of rehospitalization for older adults with heart failure and to evaluate whether that score is available for any type of heart failure.

## Methods

### Study population

We retrospectively studied 181 consecutive patients of advanced age (>65 years) with ADHF who were admitted to our hospital from January 2012 to December 2015. ADHF was defined as a gradual or rapid change in the signs and symptoms of heart failure sufficient to warrant hospitalization.^11^ Heart failure was diagnosed according to the Framingham criteria.^12^ We excluded 14 patients who were undergoing hemodialysis for chronic renal failure (*n*=3) or had a brain natriuretic peptide (BNP) level of <100 pg/mL (*n*=11).^13^

The patients’ medical records were retrospectively reviewed with regard to demographics, medical history, comorbidities, laboratory data, echocardiographic findings, medications, and clinical course. All measurements were taken at the time of hospital admission except for medications that were taken at discharge. The body mass index (BMI) was calculated as the weight in kilograms divided by the square of the height in meters. The estimated glomerular filtration rate was determined by a previously described formula.^14^ The left ventricular ejection fraction (LVEF) was calculated by the modified Simpson method.

This study complied with the standards of the Declaration of Helsinki and current ethical guidelines and was approved by the Institutional Review Board for Human Investigation of Toyonaka Municipal Hospital. Because the patients’ information was anonymized and deidentified prior to analysis, written informed consent was not obtained from the patients. However, following an extensive dialogue with the Research Ethics Committee, we publicized the study by posting a summary of the protocol (with an easily understood description) on the website of Toyonaka Municipal Hospital; the notice clearly informed patients of their right to refuse enrollment. These procedures for informed consent and enrollment were in accordance with the detailed regulations regarding informed consent described in the guidelines.

### Nutritional assessment

Nutritional indexes were calculated based on the data at admission for ADHF. The GNRI was calculated from the serum albumin level and the ratio of BW to ideal BW (IBW): IBW=22×the square of the height in meters, and GNRI=14.89×serum albumin (g/dL)+41.7×(BW/IBW). The ratio of BW to IBW was set at 1 when BW exceeded IBW.^9^ The CONUT score was calculated from the serum albumin level, total cholesterol level, and total lymphocyte count.^8^ The PNI was calculated from the serum albumin level and total lymphocyte count^10^: PNI=10×serum albumin (g/dL)+0.005×total lymphocyte count (per mm^3^).

### Endpoint and follow-up

All patients were followed up in our hospital after discharge. The endpoint of this study was unplanned hospitalization for worsening heart failure (WHF) during a period of 1 year after discharge. The decision to hospitalize a patient was made at the discretion of each primary physician.

### Statistical analysis

Continuous variables are presented as mean±standard deviation, and categorical variables are expressed as number and percentage. The unpaired Student’s *t*-test and Fisher’s exact test were used to compare differences between continuous and discrete variables, respectively. The 1-year readmission curves for heart failure were estimated using the Kaplan–Meier method, and differences between groups were compared using the log-rank test. Univariate and multivariate Cox regression analyses were employed to calculate the estimated hazard ratio (HR), with the 95% confidence interval (CI) where appropriate, and were performed to determine significant predictors of WHF for a period of 1 year. The variables were entered into a multivariate model for factors with a *p*-value of <0.05 in the univariate analysis, excluding factors closely related to nutrition scores. Receiver operating characteristic (ROC) curve analysis was performed to evaluate the prognostic accuracy of the GNRI for endpoints. A *p*-value of <0.05 was considered to indicate a statistically significant difference. Statistical analysis was performed with a standard statistical program package (JMP Pro Version 15.0; SAS Institute, Cary, NC, USA).

## Results

### Identification of most powerful predictor of WHF among three nutritional indexes

**Figure 1:**
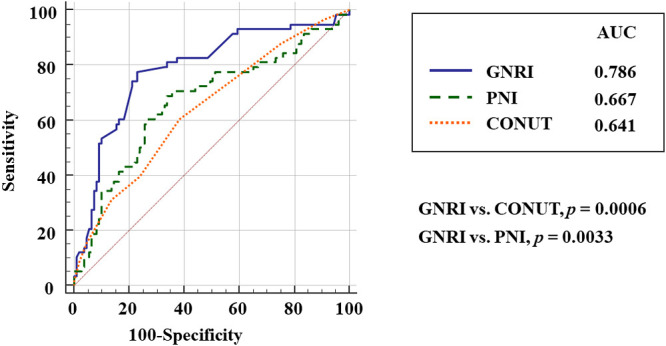
Comparison of receiver operating characteristic (ROC) curves of the geriatric nutritional risk index (GNRI), controlling nutritional status (CONUT) score, and prognostic nutritional index (PNI) for the prediction of worsening heart failure (WHF). The area under the ROC curve (AUC) is also shown.

We enrolled 181 patients who were admitted to our hospital for ADHF. We excluded 14 patients who were undergoing hemodialysis for chronic renal failure or had a BNP level of <100 pg/mL, and the remaining 167 patients were analyzed in this study. During a mean follow-up period of 280±180 days, 58 (35%) patients were readmitted with ADHF within 1 year after discharge. No patient died before hospitalization during the follow-up period. However, 13 patients died after readmission (sudden cardiac death, *n*=5; pump failure, *n*=8). We divided the patients into two groups according to the occurrence of WHF and compared these two groups. Table 1 shows the baseline characteristics of patients with and without WHF. For all analyzed patients, the mean age on admission was 81±8 years, 53% were male, the mean LVEF was 42%±16%, and 48% were classified as having New York Heart Association functional class IV heart failure. Compared with patients without WHF, those with WHF had a higher frequency of New York Heart Association class IV heart failure, higher creatinine level, and higher rate of diuretic use and a significantly lower BMI, hemoglobin level, serum albumin level, and estimated glomerular filtration rate. With regard to nutritional indexes, the GNRI and PNI were significantly lower and the CONUT score was significantly higher in patients with than without WHF. The covariate-adjusted Cox proportional hazard model showed that the GNRI, but not the PNI or CONUT score, was significantly associated with WHF (Table 2). Comparison of ROC curves among the three nutritional indexes revealed that the area under the curve of the GNRI was significantly higher than that of either the CONUT score or PNI (Figure 1). These findings suggest that the GNRI is the most powerful predictor of unplanned hospitalization for WHF among the three nutritional indexes.

### Appropriate cutoff value of GNRI for prediction of WHF

**Figure 2:**
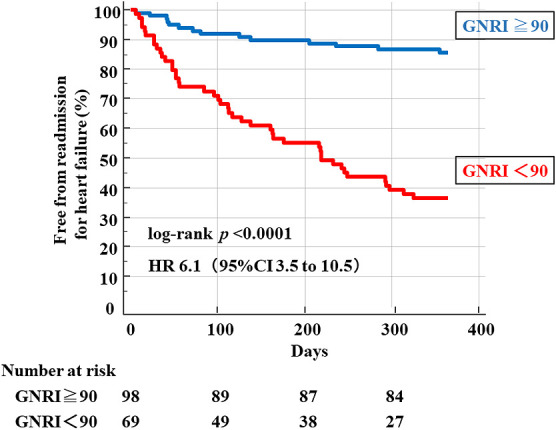
Kaplan–Meier analysis of worsening heart failure (WHF) according to the geriatric nutritional risk index (GNRI).

ROC analysis of the GNRI revealed that a GNRI of 90 was an optimal cutoff value for the prediction of WHF (area under the curve, 0.786; 95% CI, 0.716–0.846), with a sensitivity of 77.6% and a specificity of 77.1% (*p*<0.001) (Figure 1). We then divided the patients into two groups according to their GNRI score: a high-GNRI group (GNRI ≥90) and a low-GNRI group (GNRI <90). Kaplan–Meier analysis (Figure 2) revealed that patients in the low-GNRI group had a significantly greater risk of unplanned hospitalization for WHF (HR, 6.1; 95% CI, 3.5–10.5; log-rank *p*<0.0001).

### Relevance of predictive value of GNRI for WHF in various types of heart failure

**Figure 3:**
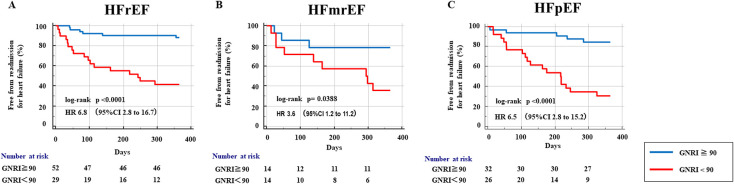
Kaplan–Meier analysis of worsening heart failure (WHF) categorized by the left ventricular ejection fraction (LVEF). (A) HFrEF, heart failure with reduced ejection fraction (<40%). (B) HFmrEF, heart failure with mid-range ejection fraction (40%–49%). (C) HFpEF, heart failure with preserved ejection fraction (≥50%).

Next, to evaluate the relevance of the cutoff value of the GNRI identified by ROC analysis in various types of heart failure for prediction of WHF, we divided the patients into three groups classified by the LVEF: heart failure with a reduced ejection fraction (HFrEF), heart failure with a mid-range ejection fraction (HFmrEF), and HFpEF. We also divided each LVEF group into two groups according to the GNRI (Table 3). Kaplan–Meier analyses showed that the low-GNRI group had a significantly greater risk of unplanned hospitalization for WHF for all types of heart failure (for HFrEF: HR, 6.8; 95% CI, 2.8–16.7; log-rank *p*<0.0001; for HFmrEF: HR, 3.6; 95% CI, 1.2–11.2; log-rank *p*=0.0388; and for HFpEF: HR, 6.5; 95% CI, 2.8–15.2; log-rank *p*<0.0001) (Figure 3).

## Discussion

This study showed that a poor nutritional status, as estimated by objective indexes, was associated with a higher incidence of unplanned hospitalization for WHF within 1 year after discharge in advanced-age patients with decompensated heart failure. Among the three nutritional indexes examined in this study, the GNRI was the most closely associated with a poor prognosis, and a GNRI of <90 was a significant predictor of unplanned hospitalization for WHF. The GNRI was associated with a poor prognosis for all types of heart failure (HFrEF, HFmrEF, and HFpEF). These findings suggest that the GNRI could be the most predictive nutritional index for unplanned hospitalization for heart failure in advanced-age patients initially admitted with ADHF.

Several reports have shown that a poor nutritional status as assessed by nutritional indexes is associated with a poor prognosis in patients with heart failure.^15,16^ A lower PNI was associated with mortality in patients with acute decompensated HFrEF or acute decompensated HFpEF.^17^ A lower GNRI was significantly associated with mortality in patients with HFpEF.^18^ A higher CONUT score was associated with an increase in hospitalization for heart failure and mortality in older patients with cardiovascular disease.^19^ These findings indicate that all three of these nutritional indexes are associated with a poor prognosis in patients with heart failure. In one study, patients with a high CONUT score had the highest HR for the risk of cardiovascular events among the three nutritional indexes in patients with chronic heart failure.^15^ By contrast, our results showed that the GNRI on admission was superior to the PNI or CONUT score for prediction of rehospitalization during 1 year after discharge in >65-year-old patients with ADHF. These findings may imply that the predictive value of a nutritional index for the prognosis of heart failure may differ according to the type or condition of heart failure (e.g., ADHF vs. chronic heart failure, HF in older vs. younger patients).

Although the reason that the GNRI was the most predictive index is not clear, it is possible that inflammatory disorders accompanying heart failure, such as infectious diseases or malignancies, may affect indexes other than the GNRI. This is because the PNI and CONUT score, but not the GNRI, take into account the number of lymphocytes, which reflects the inflammatory status. In addition, as opposed to the PNI and GNRI, the CONUT score is calculated from variables reflecting lipid metabolism. In short, this may reflect low plasma cholesterol secondary to statin therapy. Because statin therapy is still commonly prescribed for older patients, the CONUT score may not be the ideal tool for these patients. Furthermore, the GNRI is the only tool that takes into account both anthropometric factors and serum markers. The GNRI might be a better malnutrition screening tool than the CONUT score or PNI because it is multidimensional. The BNP level is the strongest predictor of a poor prognosis among patients with heart failure. However, the COACH study showed that the BNP level was lower in patients with HFpEF than in patients with HFrEF. Furthermore, for a given BNP level, the associated risks of all-cause mortality and hospitalization for heart failure are at least as high in patients with HFpEF as in those with HFrEF.^20^

Our study is the first to demonstrate that the GNRI can be predictive of the prognosis of older patients with various types of heart failure. The GNRI can reportedly predict mortality in patients with HFrEF^21^ and HFpEF.^18^ In this study, we showed that the GNRI can also be a predictor of the prognosis in patients with HFmrEF and that a single cutoff value may be applicable to predict the prognosis in older patients with HFrEF, HFmrEF, or HFpEF. However, after the patients were divided into three groups, the number of patients in each group was too small to perform adjustment with covariates. Further investigation with a larger number of patients will be necessary to show the relevance of the predictive value of the GNRI for the prognosis of HFmrEF.

Patients admitted with congestive heart failure often exhibit cardiac cachexia, which is characterized by a remarkable decline in muscular strength and physical activity because of decreases in skeletal muscle and fat.^22^ Cardiac cachexia causes a decrease in myocardial volume, changes in myocardial properties, and changes in skeletal muscle and adipose tissue, leading to a decrease in cardiac function and a poor prognosis.^23,24^ Especially in older patients with heart failure, malnutrition and age-dependent muscle decline contribute to severe dysfunction.^25^ Moreover, the nutritional status in older patients with heart failure is reportedly correlated with a poor prognosis.^26^ In fact, in the present study, the patients with a worse nutritional status (GNRI <90) had a significantly lower BMI (Table 3) and a poorer prognosis. Taken together, these findings indicate that a poor nutritional status may be a marker of resultant BW loss and a poor prognosis; therefore, detection of a poor nutritional status is potentially important for risk stratification in older patients with ADHF. Precise evaluation of the nutritional status using a reliable index is important to estimate the presence of cardiac cachexia in such patients.

Several studies have shown that early nutritional intervention decreases the rates of rehospitalization for heart failure and all-cause death.^27^ The present study showed that a low GNRI on admission is a predictor of readmission for heart failure irrespective of the LVEF. Detection of cardiac cachexia by the GNRI on admission may permit early intervention for cardiac cachexia and lead to improvement of the prognosis of older patients with heart failure.

### Study limitations

This study had several limitations. First, it was a retrospective, single-center study. Therefore, the possibility of unintentional selection bias of the study patients cannot be fully excluded. Second, the sample size was relatively small, limiting the number of covariates that could be involved in the multivariate analysis. It is possible that there were confounding factors that we could not adjust for. Thus, more convincing results may be obtained by extending the observation period and increasing the number of cases. Finally, because of the retrospective nature of the study, we evaluated the objective nutritional indexes only at admission and did not evaluate changes in the indexes during the follow-up period. Further prospective, large-scale studies are needed to address these issues.

## Conclusion

Among the three tested objective nutritional indexes, the GNRI was the most predictive for 1-year hospital readmission for heart failure in older patients with ADHF irrespective of the LVEF.

## Figures and Tables

**Table1:** Baseline characteristics of study patients with and without WHF

	All (*n*=167)	WHF (−) (*n*=109)	WHF (+) (*n*=58)	p-value
Age, years	81±8	82±8	81±8	0.4519
Male sex	53%	52%	53%	1.0000
Body mass index, kg/m^2^	23.2±3.7	23.7±3.3	22.2±4.0	0.0084
NYHA class IV	48%	41%	60%	0.0230
Ischemic	26%	24%	31%	0.3580
Hypertension	73%	73%	72%	1.0000
Diabetes mellitus	32%	33%	29%	0.7275
Dyslipidemia	37%	37%	36%	1.0000
Atrial fibrillation	48%	43%	57%	0.1049
Prior HF hospitalization	27%	28%	24%	0.5877
Laboratory data				
Hemoglobin, g/dL	12.0±2.2	12.2±2.2	11.4±2.0	0.0174
Lymphocytes, cells/mm^3^	1437±724	1429±653	1452±847	0.8425
Serum albumin, g/dL	3.44±0.36	3.54±0.32	3.26±0.36	<0.0001
Serum sodium, mEq/L	139±4	139±4	139±4	0.6540
Creatinine, mg/dL	1.17±0.61	1.08±0.55	1.34±0.69	0.0088
eGFR, mL/min/1.73 m^2^	51.4±22.0	55.3±21.9	44.0±20.3	0.0014
Total cholesterol, mg/dL	158±35	158±36	156±35	0.6606
Triglycerides, mg/dL	82±37	84±40	78±30	0.2752
C-reactive protein, mg/dL	1.1±2.2	1.0±2.1	1.5±2.3	0.1565
BNP, pg/mL	828±720	799±758	884±646	0.4681
Echocardiography				
LVEF	42%±16%	41%±17%	44%±15%	0.3342
LAD, mm	45±8	45±9	45±8	0.8879
E/e'	18.4±6.6	17.8±6.5	19.7±6.7	0.0856
Oral medications				
Diuretics	93%	90%	100%	0.0090
β-blocker	81%	84%	76%	0.2109
ACE inhibitor/ARB	84%	84%	85%	1.0000
Mineralocorticoid blocker	46%	50%	40%	0.2555
Nutritional indexes				
CONUT score	3.5±2.1	3.1±2.0	4.2±2.2	0.0013
GNRI	91.3±6.4	93.3±5.5	87.5±6.3	<0.0001
PNI	41.6±5.4	42.6±5.1	39.8±5.7	0.0018

ACE, angiotensin-converting enzyme; ARB, angiotensin II type 1 receptor blocker; BNP, brain natriuretic peptide; CONUT, controlling nutritional status; E/e', ratio of peak transmitral velocity during early diastole to peak mitral valve annular velocity during early diastole; eGFR, estimated glomerular filtration rate; GNRI, geriatric nutritional risk index; HF, heart failure; LAD, left atrial dimension; LVEF, left ventricular ejection fraction; NYHA, New York Heart Association; PNI, prognostic nutritional index; WHF, worsening heart failure. Data are presented as mean±standard deviation or percentage of patients.

**Table2:** Cox proportional hazards analyses for prediction of WHF

Variable	Univariate analysis			Multivariate analysis	
	HR (95% CI)	p-value		HR (95% CI)	p-value
GNRI	0.88 (0.84–0.92)	<0.0001		0.88 (0.84–0.92)	<0.0001
PNI	0.92 (0.87–0.97)	0.0016		—	—
eGFR	0.98 (0.97–0.99)	0.0019		0.98 (0.97–0.99)	0.0125
CONUT score	1.21 (1.07–1.37)	0.0019		—	—
Hemoglobin	0.87 (0.77–0.98)	0.0179		—	—
NYHA class IV	1.85 (1.09–3.12)	0.0225		1.81(1.01–3.26)	0.0458
Diuretics	5.96 (0.83–43.03)	0.0768		—	—

WHF, worsening heart failure; HR, hazard ratio; CI, confidence interval; GNRI, geriatric nutritional risk index; PNI, prognostic nutritional index; eGFR, estimated glomerular filtration rate; CONUT, controlling nutritional status; NYHA, New York Heart Association.

**Table3:** Baseline characteristics of study patients between lower and higher GNRI groups in various types of heart failure

Characteristic	HFrEF (LVEF <40%)				HFmrEF (LVEF 40%–49%)				HFpEF (LVEF ≥50%)		
	GNRI >90 (*n*=52)	GNRI <90 (*n*=29)	p-value		GNRI >90 (*n*=14)	GNRI <90 (*n*=14)	p-value		GNRI >90 (*n*=32)	GNRI <90 (*n*=26)	p-value
Age, years	81±8	82±8	0.6564		81±7	80±8	0.7607		82±8	80±8	0.4596
Male sex	44%	52%	0.6431		64%	57%	1.0000		59%	54%	0.7913
Body mass index, kg/m^2^	24.3±3.3	20.7±3.9	<0.0001		23.7±3.6	23.0±3.8	0.6278		24.0±2.8	22.3±3.6	0.0447
NYHA class IV	42%	55%	0.3536		29%	57%	0.2519		53%	50%	1.0000
Ischemia	31%	31%	1.0000		14%	43%	0.2087		16%	23%	0.5172
Hypertension	64%	66%	1.0000		100%	79%	0.2222		81%	69%	0.3611
Diabetes mellitus	42%	24%	0.1470		21%	29%	1.0000		31%	27%	0.7782
Dyslipidemia	46%	38%	0.4943		21%	21%	1.0000		41%	27%	0.4053
Atrial fibrillation	37%	45%	0.4865		71%	36%	0.1283		53%	62%	0.5991
Prior HF hospitalization	33%	31%	1.0000		14%	7%	1.0000		25%	31%	0.7693
Laboratory data											
Hemoglobin, g/dL	13.3±2.1	12.1±1.8	0.0106		12.2±2.1	11.4±1.4	0.2196		11.4±1.7	10.1±2.0	0.0075
Lymphocytes, cells/mm^3^	1628±723	1597±896	0.8674		1371±558	1503±958	0.6614		1300±530	1045±482	0.0629
Serum albumin, g/dL	3.65±0.26	3.20±0.23	<0.0001		3.64±0.25	3.07±0.28	<0.0001		3.68±0.29	3.11±0.26	<0.0001
Serum sodium, mEq/L	139±3	139±3	0.7411		140±3	139±3	0.7224		139±4	138±6	0.4481
Creatinine, mg/dL	1.02±0.34	1.10±0.43	0.3576		1.01±0.50	1.18±0.58	0.4141		1.32±0.79	1.43±0.91	0.6130
eGFR, mL/min/1.73 m^2^	57.9±20.6	51.2±19.0	0.1551		59.8±27.5	48.5±17.6	0.2057		44.2±21.4	44.5±23.9	0.9635
Total cholesterol, mg/dL	166±35	159±30	0.3459		152±34	151±34	0.9602		156±37	147±40	0.3709
Triglycerides, mg/dL	86±42	83±31	0.7072		79±41	83±44	0.7993		81±30	76±37	0.5257
C-reactive protein, mg/dL	0.6±0.9	1.1±1.8	0.0584		0.5±1.0	2.5±4.4	0.1113		0.8±1.6	2.2±3.1	0.0224
BNP, pg/mL	773±705	1371±921	0.0016		489±267	998±816	0.0357		543±513	775±487	0.0856
Echocardiography											
LVEF	28%±8%	26%±7%	0.3532		44%±3%	44%±3%	0.5302		61%±6%	60%±8%	0.6322
LAD, mm	45±8	43±8	0.1735		47±9	43±7	0.1678		48±11	46±7	0.3486
E/e'	18.1±7.2	19.0±7.1	0.5786		17.9±6.3	16.9±5.9	0.6811		19.0±6.7	18.9±5.7	0.9494
Oral medications											
Diuretics	89%	100%	0.0832		93%	100%	1.0000		91%	96%	0.6201
β-blocker	98%	86%	0.0528		93%	86%	1.0000		63%	58%	0.7903
ACE inhibitor/ARB	87%	93%	0.4783		86%	93%	1.0000		81%	65%	0.2310
Mineralocorticoid blocker	58%	52%	0.6463		43%	50%	1.0000		28%	39%	0.5744

ACE, angiotensin-converting enzyme; ARB, angiotensin II type 1 receptor blocker; BNP, brain natriuretic peptide; E/e', ratio of the peak transmitral velocity during early diastole to the peak mitral valve annular velocity during early diastole; eGFR, estimated glomerular filtration rate; GNRI, geriatric nutritional risk index; HF, heart failure; LAD, left atrial dimension; LVEF, left ventricular ejection fraction; NYHA, New York Heart Association; HFrEF, heart failure with reduced ejection fraction; HFmrEF, heart failure with mid-range ejection fraction; HFpEF, heart failure with preserved ejection fraction. Data are presented as mean±standard deviation or percentage of patients.

## Clinical Perspectives

Clinicians can utilize the GNRI, a simple method of evaluating the nutritional status of patients on admission in the daily clinical setting, as an index to estimate the risk of readmission of patients initially admitted for ADHF.

## Translational Outlook

Dedicated prospective outcome studies are needed to validate whether the GNRI may be predictive of the prognosis of older patients with various types of heart failure.
